# Fragile X-associated tremor ataxia syndrome rating scale: Revision and content validity using a mixed method approach

**DOI:** 10.3389/fneur.2022.977380

**Published:** 2022-09-14

**Authors:** Michelle H. S. Tosin, Glenn T. Stebbins, Christopher G. Goetz, Randi J. Hagerman, David Hessl, Melissa A. Zolecki, Peter K. Todd, Maureen A. Leehey, Deborah A. Hall

**Affiliations:** ^1^Department of Neurological Sciences, Rush University Medical Center, Chicago, IL, United States; ^2^Department of Pediatrics and the MIND Institute, University of California, Davis School of Medicine, Sacramento, CA, United States; ^3^Department of Psychiatry and Behavioral Sciences and the MIND Institute, University of California Davis School of Medicine, Sacramento, CA, United States; ^4^National Fragile X Foundation, McLean, VA, United States; ^5^University of Michigan, Ann Harbor, MI, United States; ^6^Ann Arbor Veterans Administration Healthcare System, Ann Arbor, MI, United States; ^7^University of Colorado School of Medicine, Aurora, CO, United States

**Keywords:** fragile X syndrome, fragile X tremor ataxia syndrome, FXTAS, outcome measures, severity of illness index

## Abstract

**Background:**

The original Fragile X-associated Tremor Ataxia Syndrome Rating Scale (FXTAS-RS) contained 61 items, some requiring modifications to better meet recommendations for patient-focused rating scale development.

**Purpose:**

Provide initial validation of a revised version of the FXTAS-RS for motor signs.

**Method:**

We conducted a two-phase mixed-method approach. In Phase 1, revision, we implemented a Delphi technique identifying pertinent domains/subdomains and developing items through expert consensus. In Phase 2, content validation, we conducted cognitive pretesting assessing comprehensibility, comprehensiveness, and relevance of items to FXTAS motor signs.

**Results:**

After five rounds of Delphi panel and two rounds of cognitive pretesting, the revised version of the FXTAS-RS was established with 18 items covering five domains and 13 subdomains of motor signs. Cognitive pretesting revealed adequate content validity for the assessment of FXTAS motor signs.

**Conclusion:**

The revised FXTAS-RS has been successfully validated for content and it is now ready for large-scale field validation.

## Introduction

Fragile X-associated tremor/ataxia syndrome (FXTAS) is an uncommon, genetic, movement disorder with motor and non-motor elements (1–4). Although there are currently no proven symptomatic or disease modifying treatments for FXTAS, the prompt identification of affected individuals within fragile X families and an active network of fragile X researchers have advanced the field to early clinical trials (5–10). This shift to treatment prioritization has created the critical need to develop FXTAS outcome measures that are reliable, validated, and appropriate for use in Phases II and III clinical trials.

The first version of the FXTAS Rating Scale (FXTAS-RS) was developed in 2006, containing 61 items from other scales validated for similar movement disorders, such as ataxia, Huntington's disease, essential tremor, and Parkinson's disease, which were considered to approximate the signs presented by FXTAS patients (11). At the time of its creation, the items were extracted directly from their original scales, without a designated domain, such as parkinsonism, ataxia, tremor, etc. Furthermore, items were not allocated to domains, and wording and scaling of items were not modified to meet the FXTAS-specific signs. These decisions may explain at least in part how some of the items failed to reach recommended clinimetric standards when analyzed in a population of 295 videotaped FXTAS patients (12). The study results demonstrated that many of the items needed to be revised or dropped from the scale and subjected to clinimetric phases of cognitive pretesting and final validation through large-sample field testing (12). Thus, our aim was to develop and collect patients' input on a revised version of the FXTAS-RS designed to assess FXTAS motor signs.

## Method

We conducted a two-phase mixed method approach using Delphi panel and cognitive pretesting techniques, following the Food and Drug Administration (FDA) guidance for instrument modification and content validation (13).

### Phase I–FXTAS-RS revision

Between 2020 and 2021, nine specialists from five sites of the United States (US) participated in five rounds of Delphi panel discussions. There were five neurologists, a pediatrician with expertise in FXTAS, two psychologists, and two nurses, one of them also a patient advocate and a family representative of a patient with FXTAS.

In Round 1, specialists individually listed the domains and subdomains relevant for the assessment of the FXTAS motor signs, according to their definitions and taxonomy. In Round 2, they established consensus on the set of domains and subdomains previously listed, rating each item with regards to the importance of each item in assessing motor signs in FXTAS by choosing one of three response options: 1- The domain/subdomain is not essential, 2- The domain/subdomain is useful, but not essential, and 3- The domain/subdomain is essential. Data from their responses were analyzed using the content validity ratio (CVR) of ≥ 0.75, as determined by Lawshe Table (14, 15).

In Round 3, specialists assessed whether the 61 items composing the first version of FXTAS-RS were contained in the domains of interest identified in the previous round. Then, the specialists reviewed the items with weaknesses in the previous clinimetric analyses, establishing consensus on which ones should be maintained, improved, or excluded. Data from their responses were also analyzed using the CVR, and in-depth discussion occurred until the percentage of agreement of 80% was reached, or the disagreement was consensually justified. In Round 4, specialists established agreement on the most appropriate type of response options and anchors in terms of structure, forms of use, administration, and clinimetric analysis (16, 17). In Round 5, specialists followed the FDA guidance and reviewed the draft scale and voted on the modified version, providing suggestions for adequacy regarding the scale format, instructions for use, items (order and utterances) and response options (scaling and anchors) (13).

### Phase II–content validation

Between January and February 2022, 10 stakeholders (five movement disorder specialists and five FXTAS patients) participated in cognitive pretesting activities. We included patients of either sex, ≥ 55 years of age who met diagnostic criteria for FXTAS (1, 18). We excluded medically unstable patients, those in an immediate postoperative period, or those with dementia clinically determined by the site investigator.

During the cognitive pretesting, the revised version of the FXTAS-RS was applied by a movement disorder neurologist to a patient with FXTAS and no demographic information was collected from these participants. Cognitive insights gained from participants' experiences using the FXTAS-RS were subjected to verbal protocol analysis (VPA) using the investigator's notes (19). VPA data were categorized according to comprehensibility, comprehensiveness, and relevance criteria for major or minor review. Minor revisions are edits made to the scale to address participant suggestions and comments that do not go back to another round of cognitive pretesting. Minor revisions include replacing words with synonyms, wording, punctuation, and/or word highlighting (underline, bold). Major revisions are those that require further review of the scale after revision. Major revisions include change of text structure (used to simplify writing), change in scaling and/or answer options, and/or replacement of words with change in meaning. Major and minor revisions were then incorporated into the revised version of the scale between rounds until data saturation was reached, defined by the absence of additional identified problems that required scale adjustments.

The original version of the FXTAS-RS was not presented to participants in the cognitive pretesting as a comparator.

## Results

For all assignments posted in each round of the Delphi panel, members responded to the task with written or oral responses during web-based meetings. Adherence with tasks and subsequent attendance on web-based consensus meetings was 96.3%.

In Round 1, specialists listed six domains (tremor, parkinsonism, ataxia, eye movement disorders, dystonia, and neuropathy) and 65 subdomains of observable FXTAS motor signs considered relevant to FXTAS assessment. In Round 2, specialists established consensus for five domains (all the above except for neuropathy) and 13 subdomains.

In Round 3, 24 of the 61 items composing the first version of the FXTAS-RS were excluded because they assess the same subdomain; 13 were excluded due to clinimetric weaknesses; and six were not consistent with the agreed domains/subdomains. With a focused strategy to keep the scale practical and as short as possible without sacrificing completeness, specialists established consensus to retain 18 items of the original FXTAS-RS in the revised version, covering all domains/subdomains emerging from Round 2. In Round 4, specialists agreed on ordinal (Likert type) response options with text anchors for 16 items, with the majority (*n* = 13) having five response options (ranging from 0 to 4), and an item with 4, 6, and 9 response options ranging from 0 to 3, 0 to 5 and 0 to 8 each. For these items 0 means no involvement/impairment and the largest number corresponds to severe involvement/impairment. For two items, it was agreed that the most appropriate type of response option is dichotomous, where 0 means absence of signs and 1 means presence.

**Figure 1:**
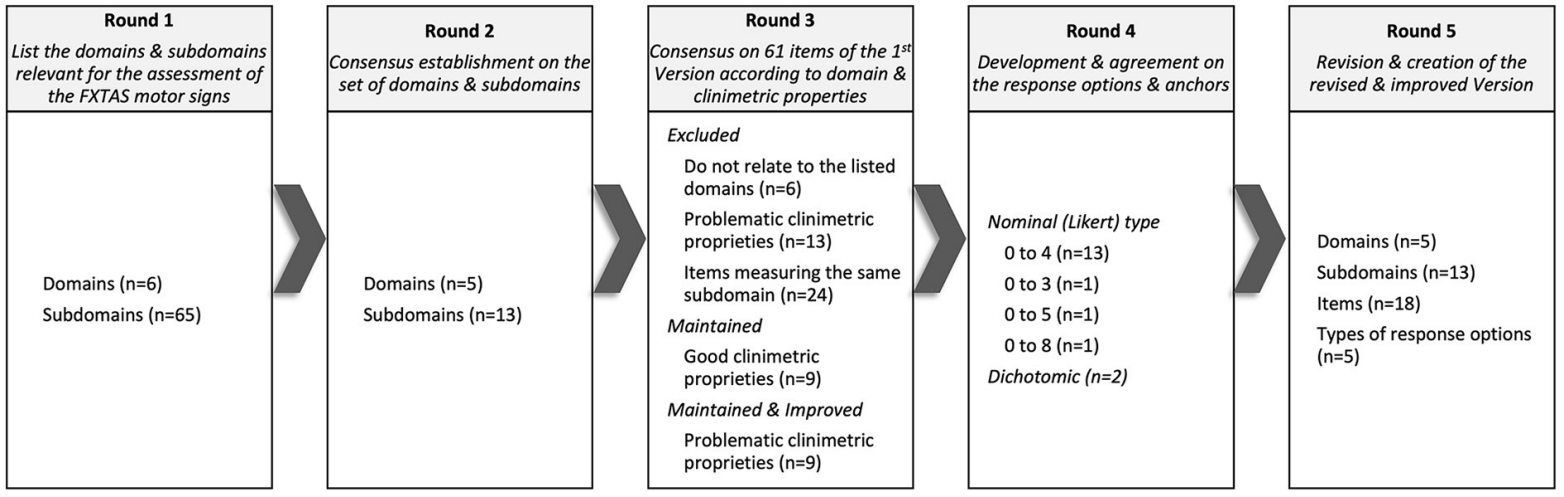
Domains, subdomains, items, and response options that reached consensus throughout the 5 rounds of the Delphi panel.

**Figure 2:**
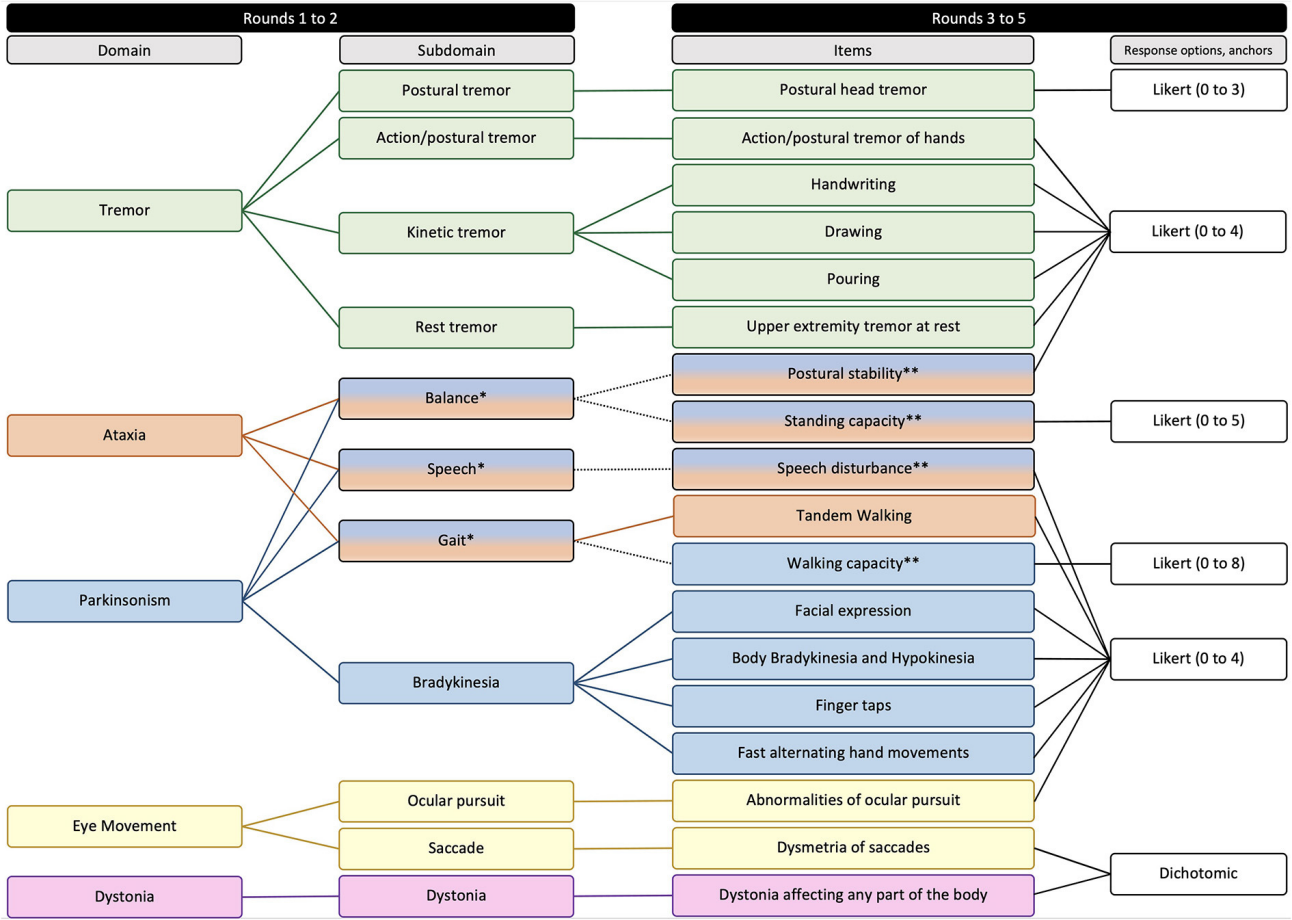
Domains, subdomains, items, and response options composing the second version of FXTAS-RS. *Subdomains represented by more than one domain. **Items represented by more than one subdomain.

In Round 5, the scale was refined for clarity, the instructions for use were created, and the final revised version of FXTAS-RS was confirmed. The Delphi process and the results obtained in each of the rounds is represented in Figures 1, 2.

For the cognitive pretesting, after two rounds no additional issues was identified, and data saturation was reached. The cognitive insights from this testing were incorporated into the revised version of the scale between rounds. All insights were categorized according to the comprehensibility criterion, as the revised version met the comprehensiveness and relevance criteria for all participants. In Round 1, of the 18 items of the FXTAS-RS, 12 received comments/suggestions from the participants, as well as the sections “Instructions for the Evaluator,” “Identification Header,” and “Ruler” (major review = 7, minor review = 13). In Round 2, of the 18 items of the FXTAS-RS, 10 received comments/suggestions from the participants, as well as the section “Instructions for the Evaluator,” and all required minor revision.

Based on provisional timing exercises in a small series of FXTAS subjects, (*N* = 5) the revised FXTAS-RS requires ~5 to 10 min to administer compared to ~20 to 30 min for the original 61-item version (See Supplemental materials for the revised FXTAS-RS).

## Discussion

The process of developing a new clinical outcome assessment is arduous, as it requires planning, construction, evaluation, and validation phases (20). The planning phase is one of the most important, since from it, the instrument content and context of use, and severity descriptions and metrics are established. The present study, using a Delphi panel and cognitive pretesting, revised and established the construct validity by assessing the patient response to a revised version of the FXTAS-RS. This methodology followed a pre-specified plan that identified and established motor impairment domains and specific items with graded anchors relevant to FXTAS motor impairment.

FXTAS case studies and studies on the natural history of FXTAS have been published describing its clinical characteristics of motor disabilities (1, 2, 18, 21, 22). From this literature, the five domains established by the Delphi panel for the revised version of the scale were based on clinical and neuropathological evidence of cortical, subcortical, and cerebellar impairment.

As with many neurodegenerative diseases, the heterogeneity of FXTAS requires a motor scale that covers the wide range and severity of motor signs. Therefore, in addition to determining domains, it is important to ensure that specific characteristics will be captured by items corresponding to a wide range of subdomains. Although FXTAS-RS originated from the unification of items from selected scales used to access motor signs in other movement disorders, in the revised version the selected items were adapted to FXTAS context. The set of procedures implement in this study allowed for the development of a specified context of use and better content validity (and, eventually, better feasibility and precision) for using the scale in clinical and research contexts. This demonstrates that the revised version of the instrument meets the clinimetric recommendations for the development of clinical rating scales (20, 23, 24). The refinement also represents a practical time savings of several minutes, an advantage especially in patients with attentional, cognitive, or physical compromise.

This study has strengths, as the implementation of the Delphi panel and cognitive pretesting with quanti-qualitative approaches, recommended for conducting clinimetric research, was used both to plan, develop and validate the content of the improved version of the proposed instrument. Despite its strengths, this study had limitations, including the panel meetings were converted from in-person to virtual format with COVID restrictions perhaps leading to less effective communication during the Delphi meetings. In addition, the study phases were conducted entirely with North American participants. However, we understand that once the North American validation phase is completed, the revised version of the FXTAS-RS will be made available for translation and cross-cultural validation, allowing the scale to be used in other global contexts.

## Conclusion

The first version of the FXTAS-RS was revised in the hopes of creating a modified more reliable and valid assessment of FXTAS motor signs. Future research will focus on the clinical validation of the revised version of the FXTAS-RS, with a goal launch in 2023.

## Data availability statement

The raw data supporting the conclusions of this article will be made available by the authors, without undue reservation.

## Ethics statement

The studies involving human participants were reviewed and approved by Institutional Review Board Institutional Review Board at Rush University Medical Center. ORA: 21082702-IRB01 Date IRB Approved: 9/20/2021. The patients/participants provided their written informed consent to participate in this study.

## Author contributions

MT, GS, and DAH organized the database and the statistical analysis plan. MT wrote the first draft of the manuscript. All authors contributed to writing the manuscript, reviewing the statistical plan, approving the submitted version, and contributed to the conception and design of the study.

## Funding

This study was funded by the Zivin Family Foundation, Steve, and Shirley Kaufman.

## Conflict of interest

The authors declare that the research was conducted in the absence of any commercial or financial relationships that could be construed as a potential conflict of interest.

## Publisher's note

All claims expressed in this article are solely those of the authors and do not necessarily represent those of their affiliated organizations, or those of the publisher, the editors and the reviewers. Any product that may be evaluated in this article, or claim that may be made by its manufacturer, is not guaranteed or endorsed by the publisher.
