# Supplementary material for: Fragile X-associated tremor ataxia syndrome rating scale: Revision and content validity using a mixed method approach
Source: Front Neurol. 2022 Sep 14;13:977380. doi: 10.3389/fneur.2022.977380 (PMC9515309; doi:10.3389/fneur.2022.977380)
Supplement: Supplementary file 1 [file Data_Sheet_1.pdf]

Supplementary Materials

FXTAS-RS Version 1.0

This scale is not the final version; further clinimetric study is planned. Before use, review the consensus rating rules on the last page. *Comments in italics were agreed upon by our group of investigators to maximize inter-rater reliability. These are not published UPDRS, CRST or ICARS guidelines.* Watch for dystonia throughout encounter to answer items 38 - 44.

1. Facial expression (UPDRS) 1. __________
0 = Normal
1 = Minimal hypomimia, could be normal "Poker Face"
2 = Slight but definitely abnormal diminution of facial expression
3 = Moderate hypomimia; lips parted some of the time
4 = Masked or fixed facies with severe or complete loss of facial expression; lips parted _ inch or more

2. Face, lips and chin tremor at rest (UPDRS) 2. Face, lips and chin_______
0 = Absent.
1 = Slight and infrequently present
2 = Mild in amplitude and persistent or moderate in amplitude, but only intermittently present
3 = Moderate in amplitude and present most of the time
4 = Marked in amplitude and present most of the time

3. Postural head tremor (Yes/No) 3. __________
0 = Not present
1 = Present

4. Abnormalities of ocular pursuit (ICARS) 4. __________
The subject is asked to follow the slow lateral movement performed by the finger of the examiner.
0 = Normal
1 = Slightly saccadic
2 = Clearly saccadic

5. Dysmetria of the saccade (ICARS) 5. __________
The index finger of the examiner is placed in the temporal visual field of the subject whose eyes are in the primary position. The subject is then asked to look laterally at the finger on the right, then to the left.
0 = Absent
1 = Bilateral clear overshoot or undershoot of the saccade
(or if there is any, even subtle, overshoot or undershoot of the saccade)

6. Speech (UPDRS) 6. __________
0 = Normal.
1 = Slight loss of expression, diction and/or volume
2 = Monotone, slurred but understandable; moderately impaired
3 = Marked impairment, difficult to understand. Unintelligible

7. Dysarthria: fluency of speech (ICARS) 7. __________

The subject is asked to repeat thee times "A mischievous spectacle in Czechoslovakia".
0 = Normal ***(uniformly slow is normal)***
1 = Mild modification of fluency
2 = Moderate modification of fluency
3 = Considerably slow and dysarthric speech
4 = No Speech

8. Dysarthria: clarity of speech (ICARS) 8. __________

0 = Normal
1 = Suggestion of slurring
2 = Definite slurring; most words understandable
3 = Severe slurring; speech not understandable
4 = No speech

9. Upper extremity tremor at rest (UPDRS) 9a. R hand________
9b. L hand________

0 = Absent.
1 = Slight and infrequently present
2 = Mild in amplitude and persistent or moderate in amplitude, but only intermittently present
3 = Moderate in amplitude and present most of the time
4 = Marked in amplitude and present most of the time

10. Action or postural tremor of hands (UPDRS) 10a. R hand________
10b. L hand________

This item is intended to rate the tremor that occurs during the trajectory of movement or during posture. The worst score of either tremor should be recorded. Tremor occurring at the endpoint of the finger-nose maneuver is not considered.
0 = Absent.
1 = Slight
2 = Mild
3 = Moderate
4 = Severe

11. Finger-to-nose test: intention tremor of the finger (ICARS) 11a. R arm________
11b. L arm________

The studied tremor is that appearing during the endpoint phase of the movement.
0 = No trouble
1 = Slight
2 = Mild
3 = Moderate
4 = Severe

12. Finger Taps (UPDRS) 12a. R hand_______
12b. L hand_______

Subject taps thumb with index finger in rapid succession with widest amplitude possible, each hand separately. Watch for the full ten seconds for degradement before scoring.
0 = Normal

1 = Mild Slowing and/or reduction in amplitude
2 = Moderately impaired. Definite and early fatiguing. May have occasional arrests in movement
3 = Severely impaired. Frequent hesitation to initiating movements or arrests in ongoing movement
4 = Can barely perform the task

13. Hand Movements (UPDRS) 13a. R hand______
Subject opens and closes hands in rapid 13b. L hand______
Succession with widest amplitude possible, each hand separately.
0 = Normal.
1 = Mild Slowing and/or reduction in amplitude
2 = Moderately impaired. Definite and early fatiguing. May have occasional arrests in movement
3 = Severely impaired. Frequent hesitation in initiating movements or arrests in ongoing movement
4 = Can barely perform task

14. Rapid Alternating Movements of Hands (UPDRS) 14a. R hand_____
Pronation-supination movements of hands, vertically 14b. L hand______
or horizontally, with as large an amplitude as possible.
0 = Normal
1 = Mild slowing and/or reduction in amplitude
2 = Moderately impaired. Definite and early fatiguing. May have occasional arrests in movement
3 = Severely impaired. Frequent hesitation in initiating movements or arrests in ongoing movement.
4 = Can barely perform the task.

15. Pronation-supination alternating movements (ICARS) 15a. R arm_______
The subject, comfortably sitting on a chair is asked to 15b. L arm_______
raise his/her forearm vertically and to make alternative movements of the hand. Each hand is moved and assessed separately.
0 = Normal
1 = Slightly irregular and slowed
2 = Clearly irregular and slowed but without sway of the elbow
3 = Extremely irregular and slowed movement with sway of the elbow
4 = Movement completely disorganized or impossible

16. Leg tremor at rest (UPDRS) 16a. R foot_______
0 = Absent 16b. L foot_______
1 = Slight and infrequently present
2 = Mild in amplitude and persistent or moderate in amplitude, but only intermittently present
3 = Moderate in amplitude and present most of the time
4 = Marked in amplitude and present most of the time

17. Leg Agility (UPDRS) 17a. R leg_______
Subject taps heel on ground in rapid succession, 17b. L leg_______
picking up entire leg. Amplitude should be about 3 inches.
0 = Normal
1 = Mild slowing and/or reduction in amplitude
2 = Moderately impaired. Definite and early fatiguing. May have occasional arrests in movement
3 = Severely impaired. Frequent hesitation in initiating movements or arrests in ongoing movement
4 = Can barely perform the task

18. Quality of sitting position (ICARS) 18. _______
Thighs together, on a hard surface, arms folded.
0 = Normal
1 = With slight oscillations of the trunk
2 = With moderate oscillations of the trunk and legs
3 = With severe dysequilibrium
4 = Impossible

19. Arising from Chair (UPDRS) 19. _______
Subject attempts to arise from a straight-back wood or metal chair with arms folded across chest.
0 = Normal
1 = Slow; or may need more than one attempt
2 = Pushes self up from arms of seat.
3 = Tends to fall back and may have to try more than one time, but can get up without help
4 = Unable to arise without help

20. Standing capacities, eyes open (ICARS) 20. ________
The subject is asked first to try and stay on one foot; if impossible to stand with feet in tandem position; if impossible to stand feet together. For the natural position, the subject is asked to find a comfortable standing position.
0 = Normal: able to stand on one foot more than 10 seconds
1 = Able to stand with feet together, but no longer able to stand on one foot more than 10 seconds.
(able to stand in tandem for more than ten seconds)
2 = Able to stand with feet together, but no longer able to stand with feet in tandem position.
3 = No longer able to stand with feet together, but able to stand in neutral position without support, with no or moderate sway.
4 = Standing in natural position without support, with considerable sway and considerable corrections.
5 = Unable to stand in natural position without strong support of one arm.
6 = Unable to stand at all, even with strong support of two arms

21. Spread of feet in natural position without support, eyes open (ICARS)
The patient is asked to find a comfortable position: 21. ________
then the distance between medial malleoli is measured.
0 = Normal (≤ 10 cm)
1 = Slightly enlarged (> 10 cm)
2 = Clearly enlarged (25 cm < spread> 35 cm)
3 = Severely enlarged (> 35 cm)
4 = Standing in natural position impossible

22. Body sway with feet together, eyes closed (ICARS) 22. ________
0 = Normal
1 = Slight oscillations ***(clearly visible and <2 cm off the midline at level of head)***
2 = Moderate oscillations (***2 -*** 10 cm at the level of the head)
3 = Severe oscillations (> 10 cm at the level of the head) threatening the upright position
4 = Immediate falling

23. Postural Stability (UPDRS) 23.________
Response to sudden posterior displacement produced by pull on shoulders while subject is erect, with eyes open and feet slightly apart. Subject is prepared.
0 = Normal
1 = Retropulsion, but recovers unaided
2 = Absence of postural response; would fall it not caught by examiner
3 = Very unstable, tends to lose balance spontaneously
4 = Unable to stand without assistance

24. Body Bradykinesia and Hypokinesia (UPDRS) 24. ________
Combining slowness, hesitancy, decreased arm swing, small amplitude, and poverty of movement in general.
0 = None
1 = Minimal slowness, giving movement a deliberate character; could be normal for some persons.
Possibly reduced amplitude

2 = Mild degree of slowness and poverty of movement which is definitely abnormal. Alternatively some reduced amplitude
3 = Moderate slowness, poverty or small amplitude of movement
4 = Marked slowness, poverty or small amplitude of movement

25. Posture (UPDRS) 25.________
0 = Normal erect
1 = Not quite erect, slightly stooped posture; could be normal for older person
2 = Moderately stooped posture, definitely abnormal; can be slightly leaning to one side
3 = Severely stooped posture with kyphosis; can be moderately leaning to one side
4 = Marked flexion with extreme abnormality of posture

Watch for dystonic posturing as subject walks.

26. Gait (UPDRS) 26. _______
0 = Normal
1 = Walks slowly, may shuffle with short steps, but no festination or propulsion
2 = Walks with difficulty, but requires little or no assistance; may have some festination, short steps or propulsion
3 = Severe disturbance of gait, requiring assistance
4 = Cannot walk at all, even with assistance

27. Walking capacities (ICARS) 27. _______
Observed during a 10-meter test including a half-turn, near a wall at about 1.5 meter.
0 = Normal
1 = Almost normal naturally, but unable to walk with feet in tandem position. ***Walk is slightly abnormally. Ignore the "unable to walk with feet in tandem position".***
2 = Walking without support, but clearly abnormal and irregular
3 = Walking without support but with considerable staggering; difficulties in half turn
4 = Walking with autonomous support no longer possible: the patient uses the episodic support of the wall for a
10-meter test
5 = Walking only possible with one stick
6 = Walking only possible with two sticks or with a stroller
7 = Walking only with an accompanying person
8 = Walking impossible, even with accompanying person (wheelchair)

28. Gait speed (ICARS) 28. _______
Observed in patients with preceding score 1-3. Preceding score 4 and up gives automatically score 4 in this test.
0 = Normal
1 = Slightly reduced
2 = Markedly reduced
3 = Extremely slow
4 = Walking with autonomous support no longer possible

29. Tandem walking (UHDRS) 29 ________
0 = Normal for 10 steps
1 = 1 - 3 deviations from straight line
2 = >3 deviations
3 = Cannot complete
4 = Cannot attempt

Do you see dystonic posturing as subject walks?
Yes No

30. Handwriting (CRST) 30. ________
Have patient write "This is a sample of my best handwriting," sign his/her name, and date. Dominant hand only.
0 = Normal
1 = Mildly abnormal. Slightly untidy. Tremulous
2 = Moderately abnormal. Legible, but with considerable tremor
3 = Markedly abnormal, illegible
4 = Severe abnormal. Unable to keep pencil or pen on paper without holding hand down with the other hand

31 – 33. Drawings (CRST)
The subject joins both points of the various drawings A, B and C without crossing the lines. Each hand is tested, beginning with the lesser involved. *The subject is instructed not to lean the hand or arm on the table.* The sheet of paper is to be fixed to the table to avoid artefacts.
0 = Normal
1 = Slightly tremulous. May cross line occasionally
2 = Moderately tremulous or crosses line frequently
3 = Accomplishes the task with great difficulty. Many errors
4 = Unable to complete drawing

	Right	Left
Drawing A	31a.	31b.
Drawing B	32a.	32b.
Drawing C	33a.	33b.

34. Drawing of the Archimedes' spiral on a pre-drawn pattern (ICARS)
The dominant hand is examined. See the figure below to calculate score. 34. _______
0 = Normal
1 = Impairment and decomposition the line quitting the pattern s slightly but without hypermetric swerve
2 = Line completely out of the pattern with recrossings and/or hypermetric swerves
3 = Major disturbance due to hypermetria and decomposition
4 = Drawing completely disorganized or impossible

35. Pouring (CRST) 35a. R hand_______
Using firm plastic cups (8cm tall). filled with water 35b. L hand_______
to 1 cm from top. The subject pours water from one cup into the other. Each hand is tested separately.
0 = Normal

1 = More careful than a person without tremor, but no water is spilled
2 = Spills a small amount of water (up to 10% of total amount)
3 = Spills a considerable amount of water (>10-50%)
4 = Unable to pour without spilling most of the water

Based on handwriting, drawing, and pouring, do you see dystonic posturing?<br><br>Yes: R arm   Neither arm<br>L arm

36. Action tremor in the heel-to-knee test (ICARS) 36a. R leg________
The test is performed in the supine position, but the head is tilted so that visual control is possible. The subject is asked to raise one leg and place the heel on the knee for 5 seconds before sliding down the anterior tibial surface. Action tremor of the heel on the knee is specifically observed. 36b. L leg________

0 = No trouble
1 = Tremor stopping immediately ***(within 1 second)*** when the heel reaches the knee
2 = Tremor stopping in less than 10 seconds ***(2 – 10 seconds)*** after reaching the knee
3 = Tremor continuing for more than 10 seconds after reach the knee
4 = Uninterrupted for tremor or test impossible

37. Knee-tibia test: decomposition of movement and intention tremor (ICARS)
Same as preceeding item. Visual control is required. 37a. R leg_______
The subject is requested to raise one leg and place the heel on the knee and then slide the heel down the anterior tibial surface of the resting leg towards the ankle. On reaching the ankle joint, the leg is again raised in the air to a height of approximately 40 cms and the action is repeated. At least 3 movements of each limb must be performed for proper assessment. 37b. L leg_______

0 = Normal
1 = Lowering of heel is continuous axis, but the movement is decomposed in several phases, without real jerks or abnormally slow
2 = Lowering jerkily in axis
3 = Lowering jerkily with lateral movements
4 = Lowering jerkily with extremely strong lateral movements or test impossible

38 – 44. Considering data from the entire tape, rate presence and severity of dystonia in each body part using the following scale:
0 = None
1 = Minimal
2 = Mild
3 = Moderate
4 = Severe

38. Face ______________
39. Voice ______________ (Spastic dysphonia)
40. Neck ______________
41. Truncal ______________
42a. Arms RUE ______________
42b. LUE ______________
43a. Legs RLE ______________
43b. LLE ______________
44. Gait ______________

FXTAS-RS Version 2.0

2022

RATING SCALE UNDER VALIDATION

FRAGILE X-ASSOCIATED TREMOR/ATAXIA SYNDROME RATING SCALE (FXTAS-RS)

MOTOR SIGNS SEVERITY

VERSION 2.0

Authored by:
Deborah A. Hall
Glenn T. Stebbins
Michelle H. S. Tosin
Maureen A. Leehey
Peter K. Todd
Randi J. Hagerman
Christopher G. Goetz
David. R. Hessl
Melissa A. Zolecki

Hall DA, Stebbins GT, Tosin MHS, Leehey MA, Todd P, Hagerman RJ, Goetz CG, Zolecki M. Fragile X-Associated Tremor/Ataxia Syndrome Rating Scale (FXTAS-RS): Motor Signs Severity. Version 2.0, 2022

ISSN:

1. FXTAS 2. Fragile X-associated Tremor Ataxia Syndrome, 3. Clinimetrics, 4. Outcome Measures, 5. Severity of Illness Index

Fragile X-associated Tremor/Ataxia Syndrome Rating Scale (FXTAS-RS): Motor Signs Severity© 2022 – licensed under CC BY-NC-ND 4.0

RATING SCALE UNDER VALIDATION

Instructions for the Evaluator

The Fragile X-associated Tremor/Ataxia Syndrome Rating Scale (FXTAS-RS) is a Clinician-Reported Outcome Assessment (ClinRO) developed to assess **signs of motor severity** usually manifested by FXTAS patients, and therefore can be used with no time limit between applications.

The FXTAS-RS assesses the severity of 18 motor signs relevant to the domains tremor, ataxia, parkinsonism, eye movements and dystonia. The severity of motor signs ranges from 0 (normal) to up to 8 (extremely severe), that must be rated during an in-person visit.

Instructions needed

- Extra pen
- 2 firm cups with water
- Ruler is available on this instrument

How to score

- Demonstrate while describing the tasks the patient must perform, but do not "pace" the patient by doing the test with him/her.
- Score all items based on the concept **"Rate what you see"**, without specific regard to whether the deficit is due to FXTAS or another co-morbidity.
- Use all information observed **throughout the examination** before setting a final score.
- If more than one option seems appropriate, **choose the more severe abnormality**.
- All individual items must have full integers, meaning not fractions and no missing rating, and they can be placed on the "Score sheet", to facilitate the sum of the total score.

The time necessary to complete the evaluation and scoring is 5 to 10 minutes.

RATING SCALE UNDER VALIDATION

FXTAS-RS© 2022 – Rating Scale under validation – licensed under CC BY-NC-ND 4.0

______	______	______	______	______
Patient identification	Site Identification/ Location	Date/ Visit	Time	Rater/ Assessor

1. Abnormalities of Ocular Pursuit

The index finger of the rater is placed about 46cm (18 inches) directly in front of the patient's eyes in the primary position. The finger is moved slowly, laterally, to a point about 30cm (12 inches) right of the primary position, then to a point about 30cm (12 inches) left of the primary position, then back to the primary position, then vertically to a point about 30cm above the primary position, then to a point about 30cm below the primary position, and then, finally, back to the primary position. Rater will note the completeness and number of saccadic intrusions that occur during the whole exercise.

Score: ___

0: Absent
1: Full pursuit, but 1 to 3 saccadic intrusions
2: Full pursuit, with more than 3 saccadic intrusions
3: Impaired pursuit; incomplete range or interrupted pursuit
4: Complete gaze palsy in any plane

2. Dysmetria of the Saccade

The index finger of each hand of the rater is placed in the patient's temporal visual fields: each index finger is about 20cm (~8 inches) lateral of the primary position. The patient is then asked, on command, to look laterally at the finger on the right, then to the left (repeat one time). Rater will rate the presence of overshoot or undershoot of the saccade.

Score: ___

0: Absent
1: Overshoot or undershoot of the saccade

3. Facial Expression

Rater observes patient's facial expression looking for signs of bradykinesia, without talking and while talking. Signs of facial bradykinesia include reduced blinking, reduced smiling, reduced movements around the mouth, and the mouth hanging open at times.

Score: ___

0: Normal
1: Slight decrease of the expression, could be normal "Poker Face"
2: Mild but decrease of the expression; lips are not parted at rest
3: Moderate decrease of the expression; lips parted some of the time
4: Severe decrease or complete loss of the expression; lips parted 0.5cm or more

4. Speech Disturbance

Speech is assessed during normal conversation. Rater will observe voice tone and volume; and diction, fluency, and speech intelligibility.

Score: ___

0: Absent
1: Suggestion of speech disturbance
2: Impaired speech, but easy to understand
3: Occasional words difficult to understand
4: Many words difficult to understand or anarthria

FXTAS-RS© 2022 – licensed under CC BY-NC-ND 4.0

CONFIDENTIAL

5. Postural Head Tremor

Rater observes presence of tremor of the head that may appear at any time during the exam, while the patient is in the sitting position.

Score: ___

0: Absent
1: Slight, l[illegible] than 1cm in amplitude
2: Moderate, 1cm to 3cm in amplitude
3: Severe, over 3cm in amplitude

6. Upper Extremity Tremor at Rest

The resting tremor will be assessed bilaterally and only the worst side will be scored. Rater observes tremor of the upper extremities that may appear at any time during the exam, including when quietly sitting, during walking, and during activities when some body parts are moving but others are at rest.

Score (worst side): ___

0: Absent
1: Slight, less than 1cm in amplitude
2: Mild, 1cm to 3cm in amplitude
3: Moderate, more than 3cm but equal or less than 10cm in amplitude
4: Severe, over 10cm in amplitude

7. Action or Postural Tremor of Hands

Patient is to stretch both arms out in front of them, in pronation (palms down). After 10 seconds, the patient is to touch the tip of rater's pen, which is placed directly in front of one outstretched arm, and then between the patient's eyes or on the nose (if tremor is severe, then instruct the subject to instead touch their knee).
Each arm is tested with the pen to nose and back three times. The movements should be slow enough to see any tremor that might not be evident with fast movement. This item is intended to rate the worst tremor that occurs during posture or during the trajectory or endpoint of movement with pen between eyes testing.

a. Right Hand: ___
b. Left Hand: ___

0: Absent
1: Slight, less than 1cm in amplitude
2: Mild, 1cm to 3cm in amplitude
3: Moderate, equal, or more than 3cm to 5cm in amplitude
4: Severe, over 5cm in amplitude

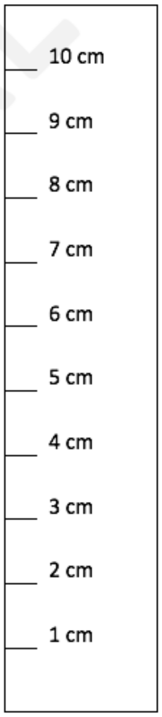

Life size ruler
US Letter
8.5 by 11 inches (215.9 by 279.4 mm)

CONFIDENTIAL

FXTAS-RS© 2022 – licensed under CC BY-NC-ND 4.0

8. Fast alternating Hand Movements

Right and left hands are rated separately. Patient sits comfortably, and if needed receives support of feet and trunk. Patient is asked to perform 10 seconds of repetitive movement of alternating pronation and supination of the hand on the thigh as quickly and accurately as possible. Precise pronation and supination movements are demonstrated by the rater. A single movement is considered to be a pronation, and another is a supination of the hand.

a. Right Hand: ___
b. Left Hand: ___

0: Normal, no irregularities
1: Slightly irregular, all single movements are clearly distinguishable
2: Mild irregularity, single movements are occasionally difficult to distinguish
3: Moderate irregularity, single movements are frequently difficult to distinguish
4: Severe irregularity of the movement

9. Finger Taps

Patient taps thumb with index finger in rapid succession with widest amplitude possible, each hand separately. Watch for the full ten seconds for decrement before scoring.

a. Right Hand: ___
b. Left Hand: ___

0: Normal
1: Slowing without decrement
2: Mild slowing with decrement
3: Moderate slowing with decrement
4: Severe slowing with decrement

10. Handwriting

Have patient write: "This is a sample of my best handwriting". Only the dominant hand is rated.

Score (dominant hand): ___

0: Normal
1: Mildly abnormal. Slightly untidy, tremulous
2: Moderately abnormal. Legible, but with considerable tremor
3: Markedly abnormal, illegible
4: Severe abnormal. Unable to keep pencil or pen on paper without holding hand down with the other hand

On the line below, please write the following sentence: *This is a sample of my best handwriting*

____

RATING SCALE UNDER VALIDATION

FXTAS-RS© 2022 – Rating Scale under validation – licensed under CC BY-NC-ND 4.0

11. Drawing

Each hand is tested, beginning with the right. The patient joins both points of the various drawings A and B without crossing the lines. The patient is instructed not to lean the hand or arm on the table. The sheet of paper is to be fixed to the table, positioned directly in front of the patient, to avoid artifacts.

0: Normal
1: Slightly tremulous. May cross line occasionally
2: Moderately tremulous or crosses line frequently
3: Accomplishes the task with great difficulty. Many errors
4: Unable to complete drawing

Drawing	Right Hand
A	
B	

DRAWING A

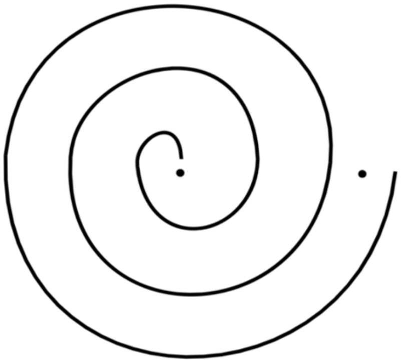

DRAWING B

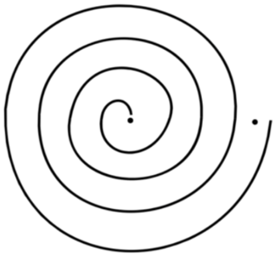

0: Normal
1: Slightly tremulous. May cross line occasionally
2: Moderately tremulous or crosses line frequently
3: Accomplishes the task with great difficulty. Many errors
4: Unable to complete drawing

Drawing	Left Hand
A	
B	

DRAWING A

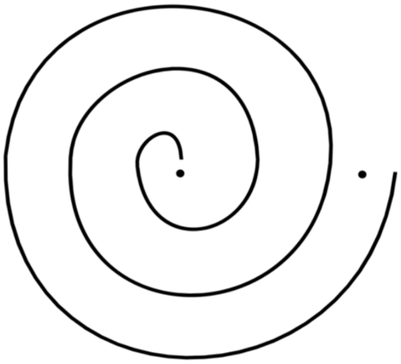

DRAWING B

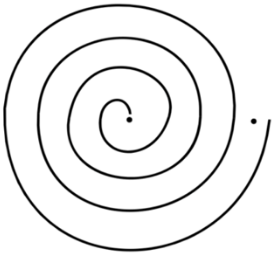

FXTAS-RS© 2022 – Rating Scale under validation – licensed under CC BY-NC-ND 4.0

12. Pouring

Each hand is tested separately and only the worst side will be scored. Use firm cups, filled with water to 1cm from top. The patient pours water from one cup into the other, keeping distance of 8cm (~3 inches) tall between the cups.

Score (worst side): ___

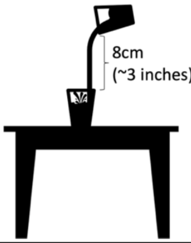

0: Normal
1: Tremor is present, but no water is spilled
2: Spills a small amount of water (up to 10% of total amount)
3: Spills a considerable amount of water (more than 10 to 50%)
4: Unable to pour without spilling most of the water

13. Standing Capacities, Eyes Open

The patient is first asked to try and stand on one foot without support for 10 seconds. If impossible, then the patient is asked to stand with the feet in tandem position without support for 10 seconds. Tandem position must be with heel touching toe, and with both feet in a straight line (neither foot is angled to the side). If impossible, then the patient is asked to stand with feet together (including front of feet) without support for 10 seconds. If impossible, then the patient is asked to stand as usual without support for 10 seconds.

Score: ___

0: Normal. Able to stand on one foot for at least 10 seconds
1: Not able to stand on one foot for at least 10 seconds, but able to stand in tandem.
2: Able to stand with feet together for 10 seconds, but no longer able to stand in tandem position for 10 seconds.
3: No longer able to stand with feet together for 10 seconds, but able to stand without support, with no or mild sway.
4: Able to stand without support for 10 seconds, but with moderate to severe sway and needing corrections
5: Unable to stand independently for 10 seconds

14. Walking Capacities

Observe patient walking 10-meters (32.8 feet), then turn and walk back, near a wall at about 1.5 meter (about 5 feet). The patient should be encouraged to walk unaided unless it is not possible/safe.

Score: ___

0: Normal
1: Walk is slightly abnormal, almost normal.
2: Walking without support, but clearly abnormal and irregular
3: Walking without support but with considerable staggering; difficulties in half turn
4: Walking with autonomous support no longer possible: the patient uses the episodic support of the wall for a 10-meter test
5: Walking only possible with one stick
6: Walking only possible with two sticks or with a walker
7: Walking only with an accompanying person
8: Walking impossible, even with accompanying person (wheelchair)

RATING SCALE UNDER VALIDATION

FXTAS-RS© 2022 – Rating Scale under validation – licensed under CC BY-NC-ND 4.0

15. Tandem Walking

Ask Patient (with shoes on) to walk in tandem, with heel touching toe, and with both feet in a straight line (neither foot is angled to the side), for 10 steps.

Score: ___

0: No deviations for 10 steps
1: 1 to 3 deviations from straight line
2: More than 3 deviations
3: Cannot complete
4: Cannot attempt

16. Postural Stability

The rater prepares the Patient by explaining the procedure, by instructing them to stand erect with feet slightly apart, and by doing a gentle practice test prior to the one to be scored. The rater will stand behind the Patient and give a forceful, quick pull back on the shoulders. This causes the Patient to need to catch themself, due to the sudden posterior displacement of their body. The rater is prepared to catch the Patient if needed. The rater counts the number of steps taken backward by the Patient.

Score: ___

0: Normal, Patient recovers with one or two steps
1: Recovers unaided after more than two steps to 5 steps
2: Recovers unaided after more than 5 steps
3: Stands safely, but with absence of postural response. Would fall it not caught by rater
4: Severely unstable, tends to lose balance spontaneously

17. Body Bradykinesia and Hypokinesia

Combining slowness, hesitancy, decreased arm swing, small amplitude, and poverty of movement in general.

Score: ___

0: Absent
1: Minimal slowness, giving movement a deliberate character; could be normal for some persons. Possibly reduced amplitude
2: Mild degree of slowness and poverty of movement which is abnormal. Alternatively, some reduced amplitude
3: Moderate slowness, poverty, or small amplitude of movement
4: Marked slowness, poverty, or small amplitude of movement

18. Dystonia

Rater must observe the presence of involuntary muscle contraction, resulting in repetitive movements or abnormal posture of the affected body part. Body areas that can be tested are eyes and upper face, lower face, jaw and tongue, larynx, neck, right and left shoulder and proximal arm, right and left distal arm and hand including elbow, right and left pelvis and upper leg, right and left distal leg and foot, and trunk.

Score: ___

0: Absent
1: Present. Which part(s) of the body? ____

CONFIDENTIAL

FXTAS-RS© 2022 – licensed under CC BY-NC-ND 4.0

Patient identification	Site Identification/ Location	Date/ Visit	Time	Rater/ Assessor

FXTAS-RS: Score Sheet

Item	Score	Comments
1. Abnormalities of ocular pursuit		
2. Dysmetria of the saccade		
3. Facial expression		
4. Speech disturbance		
5. Postural head tremor		
6. Upper extremity tremor at rest		
7. a. Action or postural tremor of right hand		
b. Action or postural tremor of left hand		
8. a. Fast alternating hand movements right hand		
b. Fast alternating hand movements left hand		
9. a. Finger Taps right hand		
b. Finger Taps left hand		
10. Handwriting		
11. Drawing A – right hand		
Drawing B – right hand		
Drawing A – left hand		
Drawing B – left hand		
12. Pouring		
13. Standing capacities, eyes open		
14. Walking capacities		
15. Tandem walking		
16. Postural Stability		
17. Body Bradykinesia and Hypokinesia		
18. Dystonia		
**Total**		

10 cm
9 cm
8 cm
7 cm
6 cm
5 cm
4 cm
3 cm
2 cm
1 cm

Life size ruler
US Letter
8.5 by 11 inches (215.9 by 279.4 mm)

RATING SCALE UNDER VALIDATION

FXTAS-RS© 2022 – Rating Scale under validation – licensed under CC BY-NC-ND 4.0

FXTAS-RS: Score Sheet

Handwriting

In the line below, please write the following sentence: ***This is a sample of my best handwriting***

____

Drawing: Right Hand

DRAWING A

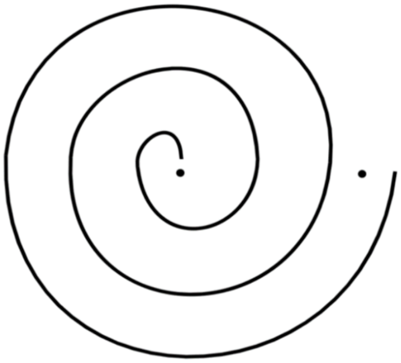

DRAWING B

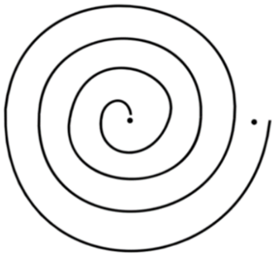

Drawing: Left Hand

DRAWING A

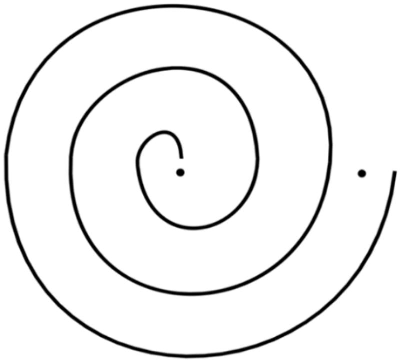

DRAWING B

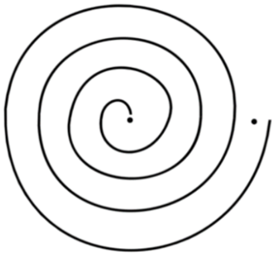

FXTAS-RS© 2022 – Rating Scale under validation – licensed under CC BY-NC-ND 4.0
